# Phylogenetic analysis of *Fasciola* spp. isolated from slaughtered cattle in KwaZulu-Natal and Mpumalanga provinces of South Africa based on the cytochrome c oxidase subunit I mitochondrial marker

**DOI:** 10.4102/ojvr.v86i1.1706

**Published:** 2019-06-18

**Authors:** Tatenda J. Chikowore, Oliver T. Zishiri, Samson Mukaratirwa

**Affiliations:** 1 Discipline of Genetics, School of Life Sciences, College of Agriculture, Engineering and Science, University of KwaZulu-Natal, Durban, South Africa; 2 Discipline of Biological Sciences, School of Life Sciences, College of Agriculture, Engineering and Science, University of KwaZulu-Natal, Durban, South Africa

**Keywords:** mtDNA, haplotype, phylogeny, interspecific, metapopulation, lymnaea

## Abstract

*Fasciola* spp. are the causative agents of fascioliasis in humans and livestock. Before the development of control and management measures, the geographical distribution of the species and patterns of infection must be considered. Because of difficulties in the phenotypic differentiation and morphometric classification of *Fasciola* spp., DNA molecular markers have become more useful for fluke differentiation and description of phylogenetic patterns. This study aimed to differentiate and describe the phylogenetic background of *Fasciola* spp. isolated from cattle slaughtered at three abattoirs in the Mpumalanga and KwaZulu-Natal provinces of South Africa. The cytochrome c oxidase I (COI) – FHCO1 (forward: 5′-TTGGTTTTTTGGGCATCCT-3′) and FHCO1 (reverse: 5′ -AGGCCACCACCAAATAAAAGA3′) – marker was sequenced from 55 Fasciola flukes that were collected from abattoirs in catchment areas of the KwaZulu-Natal and Mpumalanga provinces. *Fasciola hepatica* was demonstrated to have 100% prevalence in KwaZulu-Natal and Mpumalanga (highveld), respectively, and 76% prevalence in the lowveld (Belfast area) of Mpumalanga. Two animals from the Belfast metapopulation were co-infected with both *Fasciola gigantica* and *F. hepatica*. DNA sequence analysis of all the isolates demonstrated a sequence conservation of 0.472, nucleotide diversity of 0.082 and Tajima’s D of -1.100; however, it was not statistically significant (*p* > 0.05). Twenty-two haplotypes were identified, with 18 novel haplotypes being unique to the isolates from South Africa. Within the study samples, 12 haplotypes were isolated to a few individuals, with a haplotype diversity of 0.8957 indicating high genetic diversity. Principal coordinate analysis supported the clustering and distribution of the haplotypes, with 11.38% of the variation being attributed to coordinate 2 and 55.52% to coordinate 1. The distribution of *Fasciola* spp. has been demonstrated to be related to the distribution of the freshwater intermediate host snails, *Lymnaea* spp., as well as the relative altitude of the localities in South Africa. Information provided by this study serves as preliminary evidence for further studies on the mapping of the distribution of *F. gigantica* and *F. hepatica* in South Africa, which is key in designing control programmes for fascioliasis in humans and livestock.

## Introduction

Fascioliasis is a food-borne parasitic disease with a near global distribution and dire socio-economic effects because of human and livestock mortalities, retarded growth, reduced meat and milk production, condemnation of infected livers, animal emaciation and cost of anthelmintics for treatment (Mas-Coma et al. 2004; Mas-Coma, Valero & Bargues 2009b; Spithill & Dalton 1998). The disease occurs as a result of infection by digenean trematode species of the genus *Fasciola*. Several species have been described under the genus, with the taxonomic standing still under scrutiny and debate (Agatsuma et al. 2000; Ai et al. 2011; Hashimoto et al. 1997; Itagaki et al. 1998). However, the main causative agents of fascioliasis in livestock and humans are *Fasciola hepatica* and *Fasciola gigantica*, with hybrids being reported in areas where the distribution of the two species overlap (Ai et al. 2011; Ashrafi 2015; Walker et al. 2008; WHO 1995). Studies have reported the presence of *F. hepatica* in Africa, America, Asia, Europe and tropical islands of the Pacific (Mas-Coma, Bargues & Valero 2005; Mas-Coma et al. 2009b; Mucheka et al. 2015; Nguyen et al. 2009; Walker et al. 2008). *Fasciola gigantica*, on the contrary, has a limited geographical distribution and has been reported in Africa and Asia and some parts of southern Europe (Mas-Coma & Bargues 1997). The two trematode species are known to mainly infect humans and ruminant animals, resulting in losses in livestock production, fertility and resistance to draught in ruminants (Bernardo et al. 2011; Keiser & Utzinger 2007). Economic losses in livestock have been reported to exceed $3 billion annually, with the zoonotic aspect of the disease also making it a public health concern (Mas-Coma et al. 2009a; Spithill & Dalton 1998).

*Fasciola hepatica* and *F. gigantica* exhibit similar life cycles (Graczyk & Fried 1999; Mas-Coma & Bargues 1997). The di-heteroxenous life cycle of *Fasciola* (Andrews 1999) is dependent on Lymnaeid freshwater snails as the intermediate hosts for both *F. hepatica* and *F. gigantica* (Bargues et al. 2001). Lymnaeids have been reported to act as intermediate hosts to numerous other trematode species depending on the geographical region, ecology and snail–parasite specificity (Bargues et al. 2001; De Kock, Wolmarans & Bornman 2003; Mas-Coma et al. 2005). The life cycle of *Fasciola* includes an intermediate host snail and a definitive host with complex reproductive biology, which results in high gene flow and genetic variability within *Fasciola* spp. populations (Cwiklinski et al. 2015). Studies have shown that multiple Lymnaeid snail species may be susceptible to *Fasciola* (De Kock, Joubert & Pretorius 1989; Mas-Coma et al. 2009) and this may also contribute to potential population sub-structuring as well as development of distinct genetic clusters between different geographical localities (Beesley et al. 2017; Vilas, Vázquez-Prieto & Paniagua 2012).

Over the last few decades, reports of fascioliasis have increased, resulting in the increasing need for taxonomic clarity as well as differentiation of the two fasciolid species. *Fasciola gigantica* and *F. hepatica* overlap in their geographical distribution (Mucheka et al. 2015; Robles-Pérez et al. 2015), and this has resulted in numerous discussions and notions on the taxonomic patterns and identity of *Fasciola* spp. as well as observed intermediate forms (Agatsuma et al. 2000; Ai et al. 2011; Itagaki et al. 1998; Itagaki & Tsutsumi 1998; Nguyen et al. 2009; Periago et al. 2007). Morphological techniques, primarily used in diagnosis, take their basis on the differentiation of morphological characteristics of eggs and the adult fluke (Cuomo, Noel & White 2009; Mas-Coma et al. 2005; Thanh 2012). However, these techniques have limited sensitivity and specificity as they require skilled taxonomists who are scarcely available in many parts of the world such as South Africa. There have also been several reports of hybrids of *F. gigantica* and *F. hepatica,* which may not be morphologically differentiated from the main species (Itagaki & Tsutsumi 1998; Itagaki et al. 1998; Schweizer et al. 2007). With the increase in reports of fascioliasis and the rapid emergence of resistance to anthelmintics, further genetic analysis studies are therefore warranted. Resistance to triclabendazole to the recommended treatment dose has been reported from several parts of Eurasia (Brennan et al. 2007; Brockwell et al. 2014; Fairweather 2009; Kelley et al. 2016; Peng et al. 2009; Vilas et al. 2012), with a report of potential resistance of *F. hepatica* to treatment in a human case in the Netherlands (Winkelhagen et al. 2012). Treatment and control of fascioliasis largely depends on early diagnosis of infection, and with advances in DNA technology, application of molecular markers for species identification and genetic characterisation has become necessary in studying *Fasciola* isolates (Cwiklinski et al. 2015; Elliott et al. 2014; Mucheka et al. 2015;).

Studies have shown the utility of nuclear ribosomal markers such as ITS 1 and ITS 2 in species identification; however, mitochondrial markers are highly variable and can resolve taxonomic patterns of more closely related species and/or populations (Mucheka et al. 2015; Patwardhan, Ray & Roy 2014). The cytochrome c oxidase subunit I (COI) and nicotin adenin dinucleotide dehydrogenase (NADH dehydrogenase) subunit I (NadI) have been used as molecular markers for elucidating the phylogenetic relationships of *Fasciola* spp. and describing to an extent the genetic diversity of *Fasciola* populations (Ai et al. 2011; Hashimoto et al. 1997; Itagaki et al. 1998; Mucheka et al. 2015; Peng et al. 2009; Semyenova et al. 2006). The COI marker is widely regarded as an efficient DNA barcode and has been commonly used in species identification and differentiation of *Fasciola* spp. in recent studies in South Africa and Zimbabwe (Mucheka et al. 2015).

Against this background, the present study was aimed at identifying *Fasciola* spp. isolates collected from cattle slaughtered at abattoirs located in KwaZulu-Natal (KZN) and Mpumalanga (MP) provinces using the (mtDNA) COI region and also examining interspecies genetic diversity among the isolates using COI haplotypes.

## Materials and methods

### Study areas and collection of samples

**FIGURE 1:**
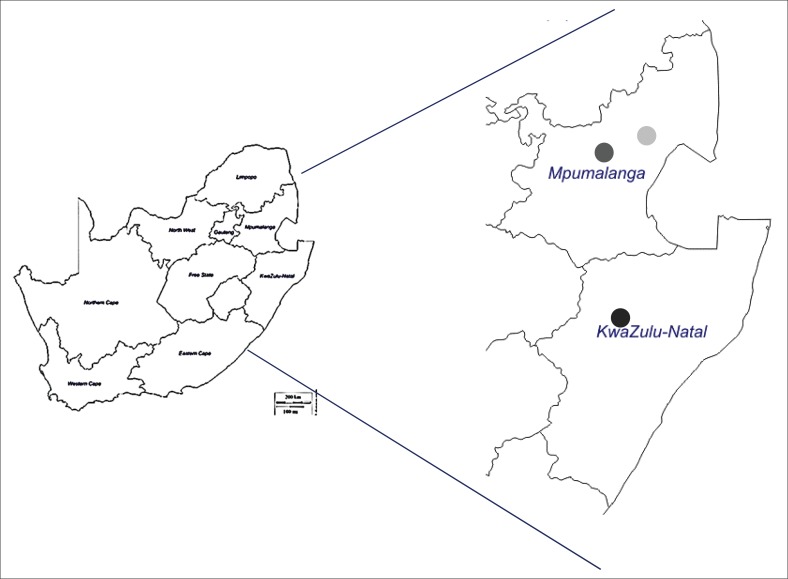
Localities were *Fasciola* spp. isolates were collected for the study. Black, Underberg (KwaZulu-Natal); grey, highveld Mpumalanga; light grey, Barberton abattoir.

A total of 55 flukes were collected from cattle at three abattoirs located in the KZN and MP provinces (Figure 1), respectively. The abattoirs served as catchment areas for cattle slaughtered in their respective areas and the exact location of origin of each animal was not determined. Twenty-one flukes were collected from cattle originating from the Swartberg and Lions River in the Underberg area of KZN (one fluke was collected from each animal), 17 were collected from the Barberton abattoir, Belfast, MP (two to three flukes collected per animal) and 17 from the Mpumalanga highveld, MP (one fluke from each animal) (Table 1).

**TABLE 1:** Identity of *Fasciola* spp. isolates from three locations in KwaZulu-Natal and Mpumalanga provinces of South Africa based on COI mitochodrial marker sequences.

Province	Origin of cattle	Number of isolates	Identity of isolates		% Prevalence of *F. hepatica*	No. of cattle with co- infection
			*F. hepatica*	*F. gigantica*		
KwaZulu-Natal	Underberg	21	21	0	100	0
Mpumalanga	Highveld	17	17	0	100	0
Mpumalanga	Lowveld (Paardeplaas, Belfast)	17	13	4	76	2

The Underberg region of KZN has a warm and cold season, with an average temperature of 14.1 °C. The average annual rainfal is 985 mm, with the altitude being 1540 m above sea level (Climate-Data 2017a). The climate and weather patterns in the Mpumalanga province depend on the topography of the specific region analysed. In Belfast, which is in the lowveld, the climate is warm and temperate and the average temperature is 13.2 °C and the average annual precipitation is 835 mm. In the highveld, the average annual temperature is 15.5 °C, with rainfall averaging 683 mm. The altitude of the province ranges from 1500 m to more than 2000 m above sea level. A decrease in temperature is noted from east to west, with the increase in altitude (Climate-Data 2017b).

### DNA extraction, amplification and sequencing

The genomic DNA was isolated from tissue from the posterior end of the fluke using the phenol–chloroform method, which uses sodium dodecylsulfate (SDS) and proteinase K to enzymatically digest proteins and non-nucleic acid cellular components. A mixture of phenol:chloroform:isoamyl alcohol (25:24:1) was used to separate lipids and cellular debris into the organic phase, resulting in isolated DNA being in the aqueous phase (McKiernan & Danielson 2017). All DNA was stored at -20 ^°^C until further analysis. Polymerase chain reaction (PCR) amplification of the partial COI region was done using the primers FHCO1 (forward: 5′-TTGGTTTTTTGGGCATCCT-3′) and FHCO1 (reverse: 5′ -AGGCCACCACCAAATAAAAGA3′) (Mucheka et al. 2015).

All PCR reactions were performed in 25 *μ*L volumes, with each reaction containing 12.5 *μ*L of DreamTaq PCR Master Mix (2X), 0.5 *μ*L per primer of the forward and reverse primers, 10.5 *μ*L of nuclease free water and 1 *μ*L of template DNA. PCR was performed using a thermocycler (Bio-Rad, Hercules, CA, United States) under the following cycling conditions: initial denaturation at 95 °C for 3 minutes (min), followed by 48 cycles of denaturation at 95 °C for 30 seconds (s), annealing at 59 °C for 30 s and 72 °C for 1 min and a final elongation step of 10 min at 72 °C, with a 10 °C hold.

Polymerase chain reaction products were run on a 1% (w/v) agarose gel, which was stained with ethidium bromide. On each gel, a molecular weight marker of 100–1000 base pairs (bp) (O’GeneRuler, Fermentas, South Africa) was used to determine the size of visible bands. The gel was viewed using the ChemiDoc™ MP Imaging System (Bio-Rad) to confirm amplification.

The unpurified PCR products were subsequently sent to the Central Analytical Facilities (CAF), Stellenbosch University (South Africa) for sequencing in the forward direction using next-generation Sanger sequencing technology. Using this technology, PCR-amplified DNA was denatured to single strands and chain-terminating dideoxynucleotides (ddNTPs) were added to the reaction. During DNA replication, the chain-terminating ddNTPs were selectively hybridized to the template strand by DNA polymerase. The chain-terminating signals were then used to determine the identity of the bases (Jamuar, D’Gama & Walsh 2016).

### Sequence analysis

BioEdit version 7.2.5 (Hall 2013) was used to edit all sequences by calling up the base peaks in the chromatogram and adjusting N, S and K substitutions to appropriate the nucleotide bases. Sequence identity was then confirmed (Table 4) using the nucleotide Basic Local Alignment Search Tool (BLAST) in the National Centre for Biotechnology (NCBI; www.ncbi.nlm.nih.gov/). Sequence alignment was performed using the MUltiple Sequence Comparison by Log-Expectation (MUSCLE) function in Mega 7, with the ends of the sequences being trimmed. The DNA Sequence Polymorphism (DnaSP) version 5 software (Librado & Rozas 2009) was used to estimate the sequence conservation, nucleotide diversity, haplotype diversity and population neutrality (Tajima’s D). Data were formatted as haploid mitochondrial DNA sequences, with nucleotide diversity being calculated using gaps or missing data considered. PAST version 3.19 (Hammer, Harper & Ryan 2001) and principal coordinate analysis (PCoA) were used for multivariate ordination analysis. The PCoA plot was generated using the Gower similarity index and c = 2 transformation exponent. Phylogenetic relationships among the populations were inferred using MrBayes version 3.2.6 (Ronquist & Huelsenbeck 2003). Four Markov chains were run for 200 000 generations to ensure a standard deviation of split frequencies less than 0.01. The print frequency was set to 1000, with a sample frequency of 10 and a burn-in period of 20 000 for Bayesian inference (BI). The haplotype network was constructed using Network 5 (Bandelt, Forster & Röhl 1999) and the median joining rooting method under default (10) weight and with epsilon (ε) set to 0.

### Ethical considerations

This study was approved by the Animal Research Ethics Committee of the University of KwaZulu-Natal (AREC/044/016PD).

## Results

### Fluke identification

**FIGURE 2:**
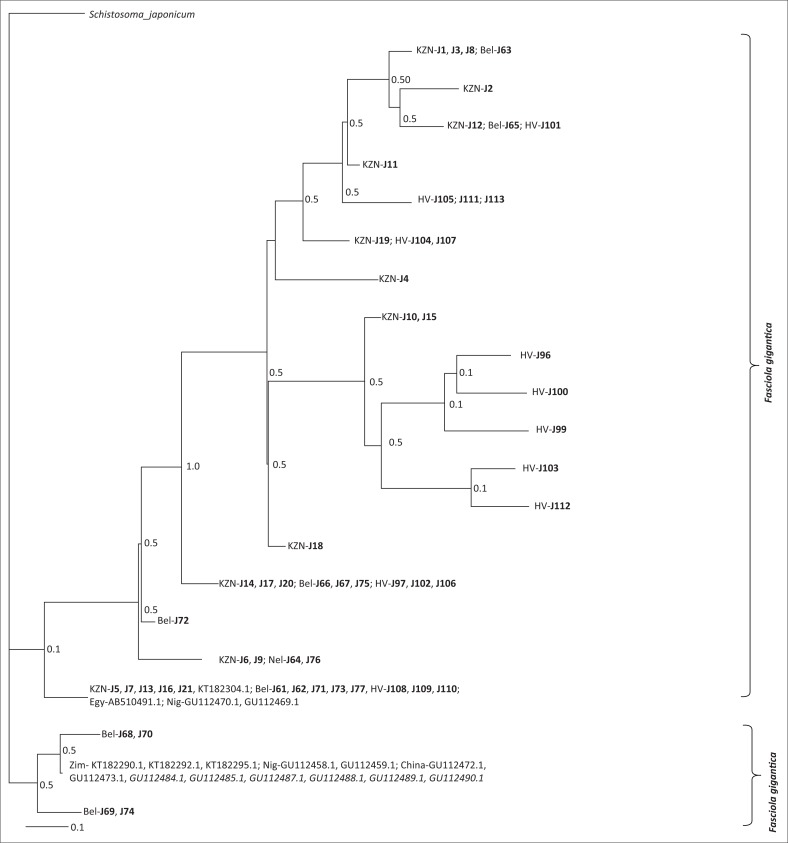
Phylogram based on haplotypes from COI sequences of *Fasciola* spp. populations from South Africa (KwaZulu-Natal and Mpumalanga provinces) and GenGank sequences using Bayesian inference. Experimental isolates are highlighted in bold and *Fasciola* spp. intermediates are shown in italics.

The trimmed COI sequences were BLAST searched against the NCBI GenBank database to obtain the closest matches, with percentage identities (Table 4) observed averaging 95% over the lowest expected values. Based on the COI sequence identities, all isolates from the Underberg region of KZN were identified as *F. hepatica,* with a prevalence of 100% (21/21), and the same results were observed with the isolates from the MP highveld (17/17). Thirteen isolates were identified as *F. hepatica* (76%; 13/17) and four isolates as *F. gigantica* (24 %; 4/17) from the Barberton abattoir in the Belfast area of MP (Figure 2). Two animals were co-infected with both *F. hepatica* and *F. gigantica* (Table 1).

### Sequence variation and diversity

The aligned KZN sequences showed a sequence conservation of 0.57, indicating moderate sequence variation although conservation was high (Table 2). The nucleotide diversity was estimated to be 0.086, indicating a low probability of difference in random sequences. Tajima’s test was performed to examine the deviation of the sequences from mutation drift equilibrium. The test yielded a Tajima’s D statistic of -0.75339, which is indicative of negative selection; however, the results were not statistically significant (*p* > 0.10). The Belfast (MP) sequences showed a sequence conservation of 0.641, indicating high sequence conservation (Table 2). The nucleotide diversity was estimated to be 0.069, also indicating low probability of random sequence difference. Tajima’s test showed a D value of -0.84641; however, it was not statistically significant (*p* > 0.10). Highveld (MP) sequences showed a sequence conservation of 0.457, indicating a higher sequence variation than conservation. The nucleotide diversity was estimated to be 0.109. A Tajima’s D of -0.608 is also indicative of negative selection although not significant (*p* > 0.10) (Table 2).

**TABLE 2:** Sequence diversity of COI in *Fasciola* isolates from the Kwa-Zulu Natal and Mpumalanga provinces.

Population	Number of haplotypes	Haplotype diversity (hd)	Nucleotide diversity (π)	Tajima’s D	Sequence conservation
Kwa-Zulu Natal	13	0.8880	0.08632	−0.75339	0.573
Mpumalanga (Highveld)	9	0.7328	0.10911	−0.60777	0.457
Mpumalanga Lowveld (Belfast)	6	0.7378	0.06901	−0.84641	0.641
**Combined**	**22**	**0.8957**	**0.08249**	**−1.10025**	**0.472**

The total aligned sequences showed a sequence conservation of 0.472, indicating moderate sequence variation with higher variable sites than conserved sites. The nucleotide diversity was estimated to be 0.082, showing low probability of random sequence difference. Tajima’s D was estimated to be -1.100 and not statistically significant (*p* > 0.05) (Table 2). The lack of significance in Tajima’s D could be attributed to the small sample size or could indicate the neutrality of the population.

### Genetic distances between haplotypes

The genetic *p*-distances between the *F. hepatica* isolates ranged from 0.01 to 0.28, while a range of 0.02–0.04 was observed for the *F. gigantica* isolates. Interestingly, the Highveld haplotypes (H_15, H_16, H_17, H_20) showed a relatively higher genetic distance to the rest of the *F. hepatica* haplotypes, with a range from 0.09 to 0.28 (Table 3). Genetic distance between haplotypes identified within the two study species ranged from 0.07 to 0.35 (Table 3), indicating a close relation between isolates of the same species and a relatively distant interspecies genetic relation.

**TABLE 3:** Estimates of genetic distances between haplotypes based on COI sequences of experimental and GenBank *Fasciola* spp. isolates.

Hap.	H_1	H_2	H_3	H_4	H_5	H_6	H_7	H_8	H_9	H_10	H_11	H_14	H_15	H_16	H_17	H_18	H_19	H_20	H_12	H_13	H_21	H_22
H_1	-	**0.01**	**0.02**	**0.01**	**0.01**	**0.01**	**0.01**	**0.01**	**0.01**	**0.01**	**0.01**	**0.01**	**0.04**	**0.02**	**0.03**	**0.02**	**0.01**	**0.03**	**0.02**	**0.02**	**0.02**	**0.05**
H_2	0.04	-	**0.02**	**0.01**	**0.01**	**0.01**	**0.01**	**0.01**	**0.01**	**0.01**	**0.01**	**0.01**	**0.04**	**0.02**	**0.03**	**0.02**	**0.01**	**0.03**	**0.02**	**0.02**	**0.02**	**0.05**
H_3	0.11	0.10	-	**0.02**	**0.02**	**0.02**	**0.02**	**0.02**	**0.02**	**0.02**	**0.02**	**0.02**	**0.04**	**0.03**	**0.03**	**0.03**	**0.02**	**0.03**	**0.03**	**0.03**	**0.03**	**0.06**
H_4	0.04	0.03	0.10	-	**0.01**	**0.01**	**0.01**	**0.01**	**0.01**	**0.01**	**0.01**	**0.01**	**0.04**	**0.02**	**0.03**	**0.02**	**0.01**	**0.04**	**0.02**	**0.02**	**0.02**	**0.05**
H_5	0.03	0.02	0.09	0.03	-	**0.01**	**0.01**	**0.01**	**0.00**	**0.01**	**0.01**	**0.01**	**0.04**	**0.02**	**0.03**	**0.02**	**0.01**	**0.03**	**0.02**	**0.02**	**0.02**	**0.05**
H_6	0.04	0.02	0.10	0.03	0.02	-	**0.01**	**0.01**	**0.01**	**0.01**	**0.01**	**0.01**	**0.04**	**0.02**	**0.03**	**0.02**	**0.01**	**0.03**	**0.02**	**0.02**	**0.02**	**0.05**
H_7	0.05	0.06	0.13	0.07	0.05	0.05	-	**0.01**	**0.01**	**0.01**	**0.01**	**0.01**	**0.04**	**0.02**	**0.03**	**0.02**	**0.01**	**0.04**	**0.02**	**0.02**	**0.02**	**0.06**
H_8	0.03	0.03	0.10	0.03	0.02	0.02	0.05-	-	**0.01**	**0.01**	**0.01**	**0.01**	**0.04**	**0.02**	**0.03**	**0.02**	**0.01**	**0.03**	**0.02**	**0.02**	**0.02**	**0.05**
H_9	0.03	0.02	0.09	0.02	0.01	0.02	0.05	0.02	-	0.01	0.01	0.01	0.04	**0.02**	**0.03**	**0.02**	**0.01**	**0.03**	**0.02**	**0.02**	**0.02**	**0.05**
H_10	0.03	0.03	0.10	0.03	0.02	0.02	0.05	0.03	0.01	-	0.01	0.01	0.04	**0.02**	**0.03**	**0.02**	**0.01**	**0.03**	**0.02**	**0.02**	**0.02**	**0.05**
H_11	0.04	0.04	0.11	0.04	0.02	0.03	0.06	0.03	0.02	0.02	-	0.01	0.04	**0.02**	**0.03**	**0.02**	**0.01**	**0.03**	**0.02**	**0.02**	**0.02**	**0.05**
H_14	0.03	0.03	0.10	0.03	0.02	0.02	0.05	0.02	0.01	0.02	0.02	-	0.04	**0.02**	**0.03**	**0.02**	**0.01**	**0.03**	**0.02**	**0.02**	**0.02**	**0.05**
H_15	0.23	0.26	0.25	0.29	0.25	0.26	0.20	0.25	0.27	0.26	0.25	0.25	-	**0.03**	**0.03**	**0.04**	**0.04**	**0.05**	**0.05**	**0.05**	**0.05**	**0.12**
H_16	0.09	0.11	0.17	0.13	0.11	0.10	0.07	0.10	0.11	0.10	0.11	0.10	0.14	-	0.02	**0.02**	**0.02**	**0.04**	**0.03**	**0.03**	**0.03**	**0.07**
H_17	0.16	0.18	0.16	0.19	0.17	0.17	0.14	0.17	0.18	0.18	0.16	0.16	0.13	0.12	-	**0.03**	**0.03**	**0.04**	**0.04**	**0.04**	**0.04**	**0.09**
H_18	0.11	0.12	0.14	0.13	0.11	0.12	0.09	0.11	0.11	0.10	0.11	0.11	0.22	0.10	0.15	-	**0.02**	**0.03**	**0.03**	**0.03**	**0.03**	**0.07**
H_19	0.06	0.05	0.09	0.06	0.05	0.05	0.07	0.05	0.05	0.05	0.06	0.05	0.22	0.09	0.14	0.08	-	**0.03**	**0.02**	**0.02**	**0.02**	**0.05**
H_20	0.22	0.21	0.20	0.23	0.21	0.21	0.23	0.21	0.22	0.21	0.21	0.21	0.28	0.24	0.23	0.16	0.16	-	**0.05**	**0.04**	**0.05**	**0.08**
H_12	0.10	0.08	0.16	0.07	0.09	0.09	0.12	0.09	0.08	0.09	0.09	0.09	0.35	0.18	0.25	0.20	0.13	0.30	-	**0.01**	**0.00**	**0.04**
H_13	0.12	0.09	0.17	0.09	0.10	0.10	0.14	0.11	0.09	0.10	0.10	0.10	0.34	0.20	0.26	0.19	0.13	0.26	0.02	-	**0.01**	**0.05**
H_21	0.12	0.09	0.17	0.08	0.10	0.10	0.13	0.10	0.09	0.10	0.10	0.10	0.36	0.20	0.26	0.21	0.14	0.31	0.02	0.04	-	**0.04**
H_22	0.35	0.31	0.39	0.30	0.32	0.33	0.36	0.33	0.31	0.32	0.33	0.33	0.62	0.44	0.51	0.44	0.36	0.51	0.28	0.31	0.27	-

Note: Genetic p-distances are shown below the diagonal and the standard error(s) (bold) are shown above the diagonal.

#### Phylogenetic analysis

**FIGURE 3:**
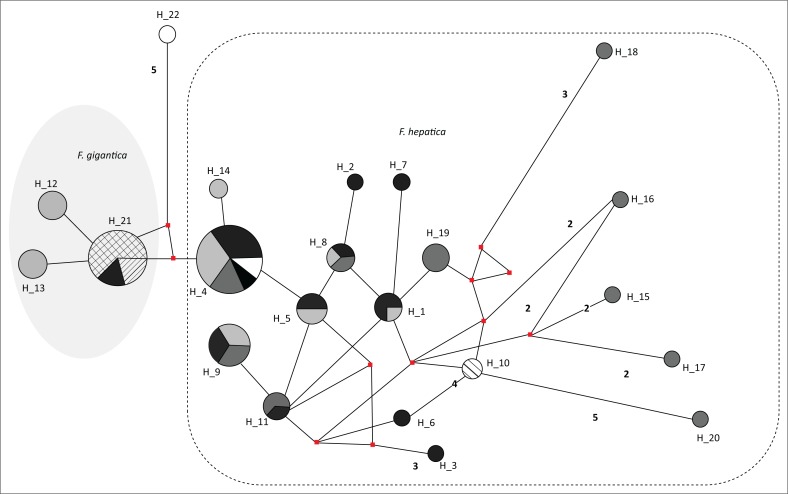
Haplotype network of *Fasciola* spp. isolates from the study and GenBank sequences. Haplotypes are represented by circles with the size of the circle indicating the number of sequences sharing that haplotype. The red squares represent median vectors. Numbers adjacent to haplotype links are the number of inferred mutational steps if more than one. Each colour is representative of the relevant geographical locality of the samples. Black fill, Niger; dark grey fill, Underberg (KwaZulu-Natal, South Africa); grey, Highveld (Mpumalanga, South Africa); light grey, Belfast (Mpumalanga, South Africa); white, Egypt; backward diagonal, Zimbabwe; diagonal cross, China; bold circle, outgroup.

The phylogenetic analysis based on the COI gene showed two main clades as shown in Figure 3. The first clade constitutes *F. hepatica* based on the identity of sequences (Table 4) from GenBank and the current study samples, which were well supported. The clade contained *F. hepatica* samples from all study sites and GenBank sequences from Egypt, Niger and South Africa (Pietermaritzburg). The second clade constitutes *F. gigantica* based on GenBank samples from Zimbabwe, Niger and China (including intermediates) and sequences from Belfast in our current study.

**TABLE 4:** Identity and locality of *Fasciola* spp. used in this study and including GenBank sequences.

Sample	Locality	Identity	% Identity	Haplotype
J1	**KwaZulu-Natal (KZN)**	*F. hepatica*	91	H_1
J2		*F. hepatica*	95	H_2
J3		*F. hepatica*	*95*	H_1
J4		*F. hepatica*	*98*	H_3
J5		*F. hepatica*	*98*	H_4
J6		*F. hepatica*	*93*	H_5
J7		*F. hepatica*	*95*	H_4
J8		*F. hepatica*	*92*	H_1
J9		*F. hepatica*	*93*	H_5
J10		*F. hepatica*	*93*	H_6
J11		*F. hepatica*	*91*	H_7
J12		*F. hepatica*	*95*	H_8
J13		*F. hepatica*	*97*	H_4
J14		*F. hepatica*	*97*	H_9
J15		*F. hepatica*	*94*	H_6
J16		*F. hepatica*	*99*	H_4
J17		*F. hepatica*	*95*	H_9
J18		*F. hepatica*	*94*	H_10
J19		*F. hepatica*	*93*	H_11
J20		*F. hepatica*	*98*	H_9
J21		*F. hepatica*	*99*	H_4
J61	**Belfast**	*F. hepatica*	*99*	H_4
J62		*F. hepatica*	*96*	H_4
J63		*F. hepatica*	*91*	H_1
J64		*F. hepatica*	*96*	H_5
J65		*F. hepatica*	*90*	H_8
J66		*F. hepatica*	*98*	H_9
J67		*F. hepatica*	*97*	H_9
J68		*F. gigantica*	*99*	H_12
J69		*F. gigantica*	*96*	H_13
J70		*F. gigantica*	*99*	H_12
J71		*F. hepatica*	*97*	H_4
J72		*F. hepatica*	*95*	H_14
J73		*F. hepatica*	*100*	H_4
J74		*F. gigantica*	*96*	H_13
J75		*F. hepatica*	*97*	H_9
J76		*F. hepatica*	*95*	H_5
J77		*F. hepatica*	*99*	H_4
J96	**Mpumalanga Highveld**	*F. hepatica*	*84*	H_15
J97		*F. hepatica*	*96*	H_9
J99		*F. hepatica*	*89*	H_16
J100		*F. hepatica*	*91*	H_17
J101		*F. hepatica*	*96*	H_8
J102		*F. hepatica*	*97*	H_9
J103		*F. hepatica*	*93*	H_18
J104		*F. hepatica*	*93*	H_11
J105		*F. hepatica*	*92*	H_19
J106		*F. hepatica*	*96*	H_9
J107		*F. hepatica*	*92*	H_11
J108		*F. hepatica*	*99*	H_4
J109		*F. hepatica*	*99*	H_4
J110		*F. hepatica*	*97*	H_4
J111		*F. hepatica*	*93*	H_19
J112		*F. hepatica*	*90*	H_20
J113		*F. hepatica*	*92*	H_19
KT182290.1	Zimbabwe (Zim)	*F. gigantica*	*N/A*	H_21
KT182292.1		*F. gigantica*	*N/A*	H_21
KT182295.1		*F. gigantica*	*N/A*	H_21
AB510491.1	**Egypt**	*F. hepatica*	*N/A*	H_4
KT182304.1	**PMB (KZN)**	*F. hepatica*	*N/A*	H_4
GU112458.1	**Niger**	*F. gigantica*	*N/A*	H_21
GU112459.1		*F. gigantica*	*N/A*	H_21
GU112470.1		*F. hepatica*	*N/A*	H_4
GU112469.1		*F. hepatica*	*N/A*	H_4
GU112472.1	**China**	*F. gigantica*	*N/A*	H_21
GU112473.1		*F. gigantica*	*N/A*	H_21
GU112484.1		*F. gigantica*	*N/A*	H_21
GU112485.1		*F. gigantica*	*N/A*	H_21
GU112487.1		Intermediate	*N/A*	H_21
GU112488.1		Intermediate	*N/A*	H_21
GU112489.1		Intermediate	*N/A*	H_21
GU112490.1		Intermediate	*N/A*	H_21
AF215860.1	**Outgroup**	*Schistosoma_japonicum*	*N/A*	H_22

#### Haplotype distribution

**FIGURE 4:**
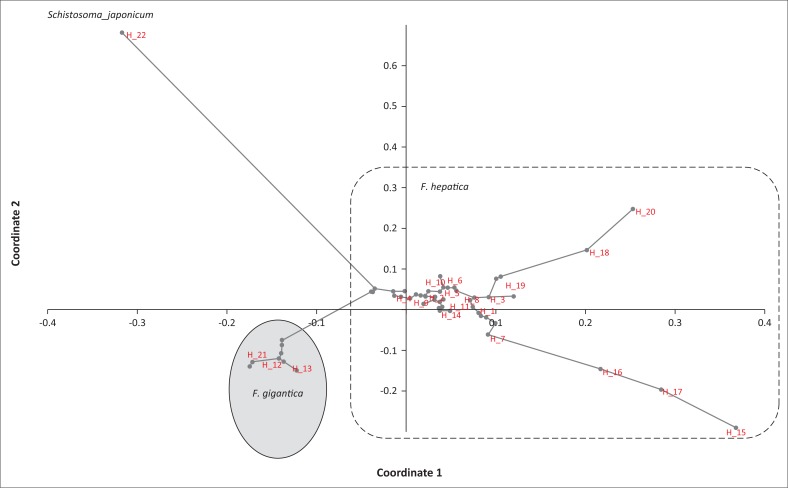
Principal coordinates analysis of *Fasciola* spp. based on the COI mitochondrial marker. Haplotypes associated with the individuals of the metapopulations of this study are indicated in red.

Analysis of experimental and GenBank *Fasciola* spp. sequences showed 22 haplotypes, with a relatively high diversity of 0.896. Haplotype H_4 was shared by *F. hepatica* sequences from KZN, Belfast, Highveld Mpumalanga, Niger and China (Figure 4). Haplotype H_21 was shown to include *F. gigantica* sequences from China, Niger and Zimbabwe and notably did not include any of the *F. gigantica* study samples, which were isolated to haplotypes H_12 and H_13 as part of the haplogroup. Haplotype H_8 and H_9 were common among the localities within the scope of this study, that is, KZN and MP, while haplotypes H_2, H_3, H_7 and H_10 were unique to KZN. Haplotypes H_1 and H_5 were shared between KZN and Highveld (MP), and haplotype H_11 contained sequences from the highveld and lowveld (Belfast) of MP. Haplotypes H_15 and H_20 contained sequences from the highveld of MP, while haplotype H_14 was isolated to the Belfast locality and haplotype H_6 was isolated to Zimbabwean isolates.

**FIGURE 5:**
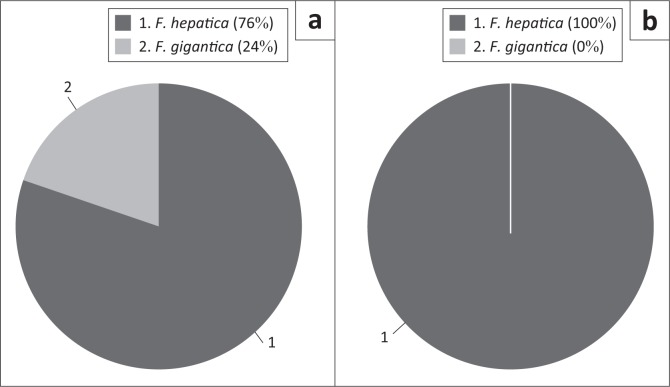
Prevalence of the *Fasciola* spp. isolated from the KwaZulu-Natal and Mpumalanga provinces of South Africa. (a) PaardePlaas, Belfaxt (Mpumalanga), (b) KwaZulu-Natal and Mpumalanga Highveld. The dark grey colour represents *F. hepatica* while the light grey colour represents *F. gigantica*.

Principal coordinate analysis supports the genetic distance and distribution of haplotypes, with the clustering of individuals being similar to the respective *F. gigantica* and *F. hepatica* haplogroups, as well as the outgroup. The PCoA plot also shows 11.38% of the variation being attributed to the left axis and 55.52% to the bottom axis (Figure 5).

## Discussion

Phenotypic identification of *Fasciola* isolates is routinely used although with difficulties (Ai et al. 2011). Molecular techniques in the differentiation of *Fasciola* isolates have been found to be effective in disease control and management (Ai et al. 2011; Beesley et al. 2017; Itagaki et al. 1998; Mucheka et al. 2015). In our study, we were able to differentiate *F. hepatica* and *F. gigantica* isolates using phylogenetic analysis of the partial COI region, which showed distinct clades of the two species. *Fasciola hepatica* isolates were restricted to KZN and the highveld area of the MP province and 76.5% to the lowveld area in Belfast. The *F. gigantica* isolates were restricted to the lowveld (Belfast) region of MP, with a 23.5% prevalence over *F. hepatica* in the same locality. This study has shown similar results, with respect to the distribution of *F. hepatica* and *F. gigantica* in the KZN and MP provinces of South Africa, to those recorded by Mucheka et al. (2015). The consistency in the results of this study with those observed in cited studies suggests the validity of the distribution patterns of *Fasciola* spp. in the study areas of South Africa.

A major driving factor of the wide distribution of *F. gigantica* and *F. hepatica* is the dependence of the life cycle on the fresh water snail intermediate host (Bargues et al. 2001; Marquardt & Demaree 1985; Mas-Coma et al. 2009). Although several snail species are used as intermediate hosts by fasciolids, *Lymnaea* spp. are notably the primary intermediate host of *Fasciola* spp. in South Africa. *Lymnaea (Galba) truncatula* is the preferred intermediate host of *F. hepatica* in Europe, and studies in South Africa have reported its distribution across the KZN and MP regions, as well as in the south of the Western Cape province (De Kock et al. 2003). *Lymnaea (Pseudosuccinea) columella* is known to be more invasive and widely distributed in South Africa and is an intermediate host for both *F. hepatica* and *F. gigantica*. Results of this study show similarities in the geographical distribution of the Lymnaeid species (De Kock et al. 2003) transmitting the two *Fasciola* species. *Lymnaea natalensis* has been identified as an intermediate host for *F. gigantica* (De Kock et al. 1989); hence, its distribution in South Africa can be related to that of *F. gigantica.* It has been reported to be present in the MP province of South Africa (De Kock et al. 1989), which could explain the presence of *F. gigantica* isolates in the Belfast region apart from the presence of *L. columella*.

The distribution of the snail intermediate hosts and the *Fasciola* spp. is dependent on several environmental factors, which, to a certain extent, are specific to the snail species. *Lymnaea truncatula* shows preference to the cooler areas and is most abundant in relatively high altitudes (De Kock et al. 2003), while *L. natalensis* shows a wide distribution in lower altitudes. *Lymnaea columella* shares a similar distribution with *L. truncatula* and has been shown to be highly invasive and adaptive as well. The present study showed high prevalence of *F. hepatica* in KwaZulu-Natal and the Highveld and Belfast of the MP province. Previous studies have shown similar results (Mucheka et al. 2015), giving an inclination to attributing the spread of *F. hepatica* in South Africa (within the study scope) to preferred habitats of the intermediate host snails *L. trancatula* and *L. columella. Fasciola gigantica* isolates were only found in the Belfast region of the MP province, which could be because of the area being suitable for breeding of *L. natalensis* and *L. columella*. The presence of both *Fasciola* spp. was noted in the Belfast region with two cattle infected with both and further studies should be conducted to determine the existence of intermediate forms of *Fasciola,* as they have been reported in regions showing species overlap, such as China (Ai et al. 2011). No intermediates were identified in the current study; however, the *F. gigantica* isolates from Belfast showed a close relationship with intermediates from China.

A link between environmental conditions and genetic diversity has been established based on Darwin’s theory of adaptation to environmental stresses as well as geographical location (Walker et al. 2011). The results of this study demonstrate low interspecific genetic diversity (π range between metapopulations = 0.06901–0.10911 and 0.07784 overall) between the flukes. This was comparatively higher than that observed by Ai et al. (2011) in a study of diversity of flukes; however, their study consisted of isolates with a wider geographical distribution. Elliott et al. (2014) recorded lower diversity in *F. hepatica* isolates from Australia. The low nucleotide diversity suggests a recent population bottleneck or founder effects as the flukes are not indigenous to South Africa but have rather spread from Eurasia (Mas-Coma et al. 2009). A relatively high haplotype diversity (*hd* = 0.8957) was also observed in this study, which is comparatively similar to that noted by Mucheka et al. (2015) in a study of *Fasciola* isolates from South Africa and Zimbabwe. Notably, only one *F. hepatica* haplotype was shared between the study isolates and GenBank-derived isolates from South Africa (KZN), Niger and China. The shared haplotype suggests a phylogenetic relationship between the isolates.

Propagated by the highly invasive *L. columella* and *L. truncatula* intermediate host snails, *F. hepatica* shows an expanding distribution globally (Mas-Coma, Esteban & Bargues 1999). *Lymnaea columella* was first reported in South Africa in 1942, with studies showing rapid expansion of its distribution across sub-Saharan Africa (Appleton 2003). *Fasciola gigantica* has been reported to be not as widely distributed as *F. hepatica* in South Africa, however (De Kock et al. 1989; Mucheka et al. 2015) unlike countries in Africa, such as Zimbabwe where it is predominant (Mucheka et al. 2015). *Fasciola gigantica* isolates from this study did not share any haplotypes with the *F. gigantica* GenBank isolates; however, they showed a relationship with only one mutational step between the haplotypes, which suggests a distant relation between the isolates. The isolates forming part of the *F. gigantica* haplogroup could share a distant maternal lineage with geographical isolation as well as microevolution contributing to diversity. *Fasciola gigantica* is reported to have spread to Africa earlier than *F. hepatica* (Mas-Coma et al. 2009) which could influence the rate of divergence of the populations isolated in South Africa because of the reported short time of haplotype change in fasciolids (Walker et al. 2011). The high haplotype diversity is also indicative of a recent population expansion, which can be attributed to the ability of fasciolids to adapt to a wide range of environmental conditions (Mas-Coma 2004; Mas-Coma et al. 2005). Climate change and human activities have been cited as major contributing factors of the transmission and spread of fascioliasis (Afshan et al. 2014; Mas-Coma et al. 2009). Life cycles of both the liver flukes and their intermediate host snails are highly dependent on geo-climatic parameters, with emphasis on temperature, rainfall and altitude (Mas-Coma 2004). Variations in climatic conditions can result in the variations in genetic diversity of the liver fluke between populations, displaying spatial and temporal separation (Walker et al. 2011). Studies have shown that husbandry and management of farms influence the movement of the definitive hosts, which, in turn, affects the population genetic diversity of the parasites (Beesley et al. 2017; Mas-Coma et al. 2005; Semyenova et al. 2006). Currently, the authors could not find conclusive studies on patterns of movement of cattle and other livestock in South Africa.

## Conclusion

Although an increase in the importance of facioliasis as a zoonotic disease has been observed, the paucity of information in Southern Africa on the phylogenetic relationships as well as genetic diversity of *Fasciola* spp. is evident. This study has demonstrated the presence of *F. hepatica* in both the KZN and MP provinces of South Africa, confirming a recent observation by Mucheka et al. (2015) with *F. gigantica* only observed in the lowveld of the MP province. *Fasciola hepatica* was shown to be prevalent in the two provinces studied and shows similarity to studies by Mucheka et al. (2015). A link between the distribution of *Fasciola* spp. and that of the Lymnaeid intermediate host snails has been established in the literature (Mas-Coma et al. 2009; Mucheka et al. 2015), giving an inclination to attributing the fasciolid distribution to that of the snail intermediate hosts. The mitochondrial COI marker showed high genetic diversity between the study populations; however, further studies using more variable and neutral markers, such as microsatellites, are required to elucidate population genetic structuring. Future research should be focused on elucidating the phylogeography of *Fasciola* spp. in other provinces of South Africa, as well as the parasite-intermediate host snail interactions. This study has added to the current knowledge regarding the distribution of *Fasciola* spp. in the KZN and MP provinces of South Africa. The study was however limited by sample size and the geographical distribution of sampling sites, which could both be increased to further elucidate the genetic structuring and distribution of *Fasciola* on a wider scope.
